# Face-to-face individual counseling and online group motivational interviewing in improving oral health: study protocol for a randomized controlled trial

**DOI:** 10.1186/s13063-015-0946-0

**Published:** 2015-09-18

**Authors:** Xiaoli Gao, Edward Chin-Man Lo, Colman McGrath, Samuel Mun-Yin Ho

**Affiliations:** Dental Public Health, Faculty of Dentistry, The University of Hong Kong, 3/F, Prince Philip Dental Hospital, 34 Hospital Road, Sai Ying Pun, Hong Kong; Department of Applied Social Sciences, City University of Hong Kong, Kowloon, Hong Kong

**Keywords:** Motivational interviewing, Adolescents, Health behaviors, Oral health, Dental caries, Periodontal diseases, Randomized controlled trial

## Abstract

**Background:**

Motivational interviewing (MI) has great potential in changing health-related behaviors. In addition to delivery in face-to-face individual counseling, MI can be delivered through online groups, a method that is particularly appealing to adolescents and may offer several benefits. This randomized controlled trial compares the effectiveness of prevailing health education (HE), face-to-face individual MI and online group MI in improving adolescents’ oral health behaviors (diet and toothbrushing) and in preventing dental caries and periodontal diseases.

**Methods/Design:**

In each of Hong Kong’s main districts (Hong Kong Island, Kowloon and the New Territories), three secondary schools will be recruited and randomly assigned to three groups (HE, face-to-face individual MI, and online group MI). A total of 495 adolescents (aged 12 to 13 years) with unfavorable oral health behaviors (“snacking twice or more a day” and/or “brushing teeth less than twice a day”) will be recruited: 165 in each group. Two dental hygienists will be trained to deliver the interventions. HE will be provided through an oral health talk. Participants in the “face-to-face individual MI” group will join a one-on-one counseling session. For “online group MI,” participants will form groups of 6 to 8 and join a synchronous text-based online counseling session. At baseline and after 6, 12 and 24 months, clinical outcomes (caries increment and gingival health) and oral health self-efficacy and behaviors (toothbrushing and snacking) will be recorded through an oral examination and a questionnaire, respectively. Effectiveness of the interventions will be evaluated and compared. The primary outcomes will be the “number of new carious surfaces” and “gingival bleeding score” (% of surfaces with gingival bleeding). The secondary outcomes will be changes in oral health self-efficacy and behaviors (toothbrushing and snacking frequencies). A preliminary economic evaluation and a process evaluation will be included to analyze the cost of the interventions and the interactions in MI sessions.

**Discussion:**

Since online group MI is expected to be more convenient, accessible, and time efficient, it might address the practicality issues and pave the way for the application of MI in dental practice. The findings will assist public health workers and dental practitioners to choose effective and viable approaches in delivering behavioral interventions. Since unhealthy diet and poor personal hygiene are common risk factors accountable for many systemic diseases, the intervention scheme identified in this study can also contribute to advancing general health.

**Trial registration:**

The HKU Clinical Trial Register #HKCTR-1852 (registered on 13 November 2014).

## Background

Common oral diseases such as dental caries (tooth decay) and periodontal diseases (gum diseases) are highly prevalent and afflict a large proportion of the world’s population [1]. The treatment of these diseases and their complications imposes a heavy financial burden on families and the society [2]. Like other chronic diseases, dental caries and periodontal diseases are highly determined by one’s “lifestyles” and are preventable if one can adopt healthy dental behaviors [1]. Two oral health self-care behaviors, namely brushing teeth twice a day and avoiding frequent snacking, are considered central to the protection of oral health [3].

Prevailing health education (HE) focuses on disseminating information and giving normative advice. A typical consultation is often an exercise in overt persuasion. However, what appears to be a convincing line of reasoning to the professionals may not be of interest to the clients or may even result in the clients’ resistance to change [4]. The insufficiency of HE in promoting oral health has been documented [5]. Although oral health knowledge can often be improved, such knowledge gain seldom translates into sustained changes in behaviors [5].

Addressing the insufficiency of HE, a person-centered counseling approach, motivational interviewing (MI), has been developed [6]. As a collaborative, goal-oriented style of communication, MI explores a client’s ambivalence and elicits client’s intrinsic motivation for and commitment to behavioral changes within an atmosphere of acceptance and compassion [4]. Its effectiveness in changing a broad range of behaviors, such as lack of physical exercise, unhealthy diet and poor compliance to medication, has been reported [7]. Motivational interviewing has been introduced into changing oral health related behaviors [8] and has demonstrated its potential in preventing early childhood caries [9, 10] and in treating adult patients with periodontitis [11, 12]. Nevertheless, its effectiveness in changing adolescents’ dental behaviors and protecting their oral health is yet to be investigated. Since adolescence is a critical stage when health habits are reinforced and perpetuated, interventions at this age are essential for combating oral diseases over a lifetime [3]. The latest oral health survey in Hong Kong has shown that 32 % of the adolescents did not brush teeth twice a day and that 36 % snacked twice a day or more often [13]. These data point to the need for behavioral interventions in this age group.

MI has been typically delivered through face-to-face individual (one-on-one) sessions, but this is not the only possible mode of delivery [4]. MI can also be adapted for delivery via other media (telephone, mails, Internet etc.) [4] or in a group format [14]. Online groups are considered promising vehicles for delivering MI to adolescents [15, 16]. Internet use has grown exponentially and has become an appealing channel through which young people interact with the world [17]. Meanwhile, the sensitivity of youth to their peers makes group work a powerful strategy for health intervention [14, 18]. More than just an alternative delivery mechanism, online group MI may offer several benefits that face-to-face individual MI cannot provide. For example, (a) it connects isolated individuals to a collaborative group where interactions take place between facilitator and participants, and also among participants themselves [14, 15]; (b) members can be inspired by others’ ideas and perspectives, which broaden the way they think about their lives’ possibilities and serve as a catalyst for them to move toward change [19]; (c) members often benefit from the support and experience of others in similar situations, gain hope and confidence that make their challenges more manageable, and discover their own path forward [19]; (d) online counseling offers a high level of anonymity, reduces embarrassment and stigma, and facilitates members’ openness and self-disclosure [20, 21]; (e) online sessions are convenient, allow members to access within the comfort of their own home, and have the potential to involve those hard to reach people [21]; and (f) online group MI, serving several members at once, are likely to be time-efficient and cost-effective, and may be a more practical approach [22].

Two pilot studies have been carried out to test online group MI among 20 adults with obesity [23] and 26 adolescent smokers [15]. Both studies supported the acceptance and feasibility of online group MI and provided preliminary evidence on its usefulness. Online group MI helped adult participants reduce weight by 2.6 kg on average in 8 weeks, reduced the amount adolescents smoked per day (4.4 to 2.4; *P* = 0.023), and increased their intention to quit (*P* = 0.038). Both pilot studies were uncontrolled, single-group trials with a small sample size and self-reported outcomes. To further test the effectiveness of online group MI, a randomized controlled trial was conducted among 136 adolescent smokers [16]. Adolescents who participated in the online group MI (n = 77) were significantly more likely than controls (n = 59) to report abstinence from smoking, fewer smoking days, and a reduction in the amount smoked after 3 months (all *P* < 0.05). Face-to-face individual MI was not, however, included in the comparison. While these early reports suggest the promises of online group MI, this mode of delivery has not been introduced to and tested in changing oral health behaviors.

This study aims to evaluate and compare the effectiveness of prevailing health education, face-to-face individual MI, and online group MI in enhancing adolescents’ oral health self-efficacy, improving their oral health behaviors (toothbrushing and snacking) and preventing common oral diseases (dental caries and periodontal diseases). The null hypothesis is that there is no difference in the effectiveness of the three interventions in changing adolescents’ oral health self-efficacy and behaviors and in preventing dental caries and periodontitis.

## Methods/Design

### Sample size calculation

The primary outcomes of this study are the “number of new carious surfaces” (∆DMFS) and “gingival bleeding score” (% of surfaces with gingival bleeding). An effect size of 0.4 (based on means) is considered clinically significant for both variable [24]. Based on a significance level of 5 % and a targeted statistical power of 80 %, 132 participants in each group will be required. Allowing for a 20 % attrition rate, 165 participants would need to be recruited into each of the three study groups. The total sample size will be 495.

### Participant recruitment and randomization

CONSORT guidelines [25] will be followed in the design, implementation and reporting of this randomized controlled trial. The protocol of this study was reviewed, and ethical approval was granted by the institutional review board of the University of Hong Kong and the Hospital Authority Hong Kong West Cluster (IRB #UW14-142).

To be eligible to join this study, a participant should (a) be a Form-1 or Form-2 student (aged 12 to 13 years) in a participating school, (b) have unfavorable oral health behavior(s) (that is, be a student who needs intervention), and (c) have Internet access via computers or mobile devices (smart phone, iPad etc.). Unfavorable oral health behaviors are defined as “snacking twice or more a day” and/or “brushing teeth less than twice a day.” A student will be excluded if he/she (i) is unable to cooperate in the related procedures due to severe health conditions, (ii) cannot speak Cantonese or (iii) cannot read and type Chinese.

This study will be conducted in nine secondary schools; three in each of Hong Kong’s three main districts (Hong Kong Island, Kowloon and the New Territories). All schools are co-ed schools, both sexes are included, and there is a balance in sexes across groups. The three schools in each district will be randomly allocated by the drawing of lots in sealed envelopes to one of three groups: (1) HE, (2) face-to-face MI or (3) online group MI. The group allocation will be done by a researcher not involved in delivering the intervention and assessing the outcomes.

All Form-1 and Form-2 students in the participating schools will be approached. Parental written consent and student’s assent will be obtained on their participation. Eligible participants will be identified through a standardized questionnaire on students’ demographic information, current snacking and toothbrushing habits, access to the Internet and language proficiency. The recruitment process will be monitored to ensure the characteristics of participants across the three groups are balanced with regard to their socio-demographic profile (sex and parental education level) and baseline behaviors (snacking and brushing). In the three schools allocated to each intervention group, 165 participants will be recruited; 55 from each school. The total number of participants will be 495 for the whole study.

### Oral health behavioral interventions

In the HE group, prevailing oral health education will be delivered through a 20-minute oral health talk given by a dental hygienist to the whole school. Aided by PowerPoint presentation and mouth models, the talk will cover several topics: (a) importance of oral health and the impacts of oral diseases on one’s daily life (physical, psychological and social well-being); (b) common oral diseases (dental caries and periodontal diseases) and their causes; and (c) tips (healthy diet, toothbrushing, etc.) for protecting oral health. There will be time for students to raise questions, which will be answered by the dental hygienist.

Each participant in the “face-to-face individual MI” group will join a one-on-one MI counseling in a quiet room provided by the school. Two dental hygienists will be trained in using MI to elicit behavioral changes. The training includes both theoretical and practical sessions and will be delivered by a health psychologist who is experienced in teaching MI in a healthcare setting. A hygienist will proceed to contact participants only when he/she demonstrates satisfactory competence as evaluated by the Motivational Interviewing Treatment Integrity (MITI) scale [26]. The counseling will follow the MI spirits (collaboration, acceptance, evocation and compassion), core skills (open questions, reflection, affirmation, and summary), and processes (engaging, focusing, evoking, and planning) elaborated by the MI founders [4]. A typical MI session lasts for 20 to 30 minutes and contains the following elements: (i) the counselor begins with establishing rapport, getting the participant to talk and engaging him/her in sharing his/her own thoughts about oral health; (ii) by asking open questions, attentive listening and reflection, the counselor will bring the conversation to a more focused and deeper stage, thereby identifying the discrepancy between the participant’s present behaviors and personal goals; (iii) the counselor will try to elicit and reinforce the participant’s own talk of change (desire, ability, reason, need, commitment, action and taking steps) and affirm his/her positive intentions, personal strength, efforts etc.; and (iv) if the participant is ready to set a plan, the counselor will explore what change he/she would like and feels confident to try, discuss the barriers and risky scenarios and explore possible solutions.

For online group MI, participants who share the same unfavorable behavior(s) (that is, infrequent toothbrushing, snacking, or both) will form a group of six to eight [14] and join an online MI session led by a dental hygienist. Through an online chat room created in a secure server, the synchronous (real time) text-based online session lasts for 30 to 45 minutes and follows the MI principles and group facilitation strategies. The session starts with a simple welcome and orientation, so that members become acclimated to the group setting and have an agreement on the topics of focus for the group. During the *engagement* phase, the counselor helps members become comfortable with participating and focuses on facilitating early group process on members’ issues and concerns. As the group moves into the *exploring perspectives* phase, the counselor helps deepen members’ interactions as they explore their individual viewpoints and situations together. During the *broadening perspectives* phase, interactions continue to deepen and broaden as the counselor encourages members to consider new possibilities, to allow buried hopes to reemerge and to take others’ perspectives into consideration. During the *moving into action* phase, the counselor guides members to develop specific change plans, to delineate steps toward their goals, to identify high risk situations (for example, outings and social functions) and environmental factors (for example, the temptation of fast food and unhealthy cooking in their families), and to explore possible solutions transferable to their daily lives. To close the session, the counselor summarizes the perspectives gained (or abandoned) and the experiences shared, highlights any messages for members to take with them and links the themes back to the large process of change that members are undertaking. During the whole session, the counselor engages members as a group rather than as a collection of individuals. While maintaining an individualized, client-centered focus, the counselor remains attuned to group dynamics and builds group cohesion and momentum by linking members’ interest, perspectives, experiences and concerns. The counselor allows the group to unfold at a comfortable and unpressured pace and finds a balance between providing structure and allowing spontaneity.

The face-to-face MI session will be audiotaped, and the text conversation in the online MI groups will be downloaded. Both will be periodically reviewed, and 15 % of the sessions will be coded using MITI to assess the MI fidelity. In both of the MI groups, each participant will only join one counseling session. Two follow-up telephone calls will be made in a month after the MI session to assist the participant’s preparation for change, encourage him/her to start, and discuss difficulties and possible solutions. To maintain the behavioral change and avoid relapse, participants will be contacted through the telephone three more times up to 6 months after the initial contact [9, 10]. The timeline and purposes of the follow-up telephone calls are shown in Table 1.

**Table 1:** Intervention activities for both motivational interviewing (MI) groups

Activity	Time	Goals
Initial counseling (X1)	Start of study	Establish rapport, discuss need and options, use strategies that structure and elicit change and set goals
Follow-up telephone calls (X2)	2 weeks and 1 month after initial counseling	Assist preparation, encourage start and solve problems
Maintenance telephone calls (X3)	2, 4 and 6 months after initial counseling	Promote maintenance, avoid relapse and help re-establish change, if needed

### Outcome measures

The primary outcomes will be the “number of new carious surfaces” (∆DMFS) and “gingival bleeding score” (% of surfaces with gingival bleeding), whereas secondary outcomes will be changes in oral health self-efficacy and behaviors (Table 2).

**Table 2:** Outcome measures

Outcomes	Variables	Codes	Source of information
Secondary outcomes			
Change in self-efficacy^a^			
Snacking	Views on the statements “I can control snacks to once a day or less when … (under several scenarios)”	(1) Not at all; (2) A little of the time;	Questionnaire
		(3) Sometimes; (4) Most of the time;	
		(5) All the time	
Toothbrushing	Views on the statements “I can brush my teeth twice a day when … (under several scenarios)”	1) Not at all; (2) A little of the time;	Questionnaire
		(3) Sometimes; (4) Most of the time;	
		(5) All the time	
Change in behaviors			
Snacking	Frequency of snacking per day	(1) None; (2) Once; (3) Twice;	Questionnaire
		(4) 3 times; (5) 4 times or more	
Toothbrushing	Frequency of toothbrushing per day	(1) Less than once; (2) Once;	Questionnaire
		(3) Twice; (4) 3 times or more	
Primary outcomes			
Gingival health	Gingival bleeding score (% of tooth surfaces with gingival bleeding)	Continuous	Oral examination
Caries increment	Number of new carious surfaces	Continuous	Oral examination
	Presence/absence of new carious surfaces	Nominal	Oral examination

^a^Six scenarios will be used, namely “when feeling down in the dumps,” “when busy and overloaded,” “when tired,” “when on a vacation,” “when it means stopping doing something that is enjoyable” and “if there is a slip up, will the subject resume controlling”

At baseline and after 6, 12 and 24 months, participants will be examined by a trained dentist calibrated against an experienced oral epidemiologist. The examiner will be blinded to the group allocation of each school. Dental caries will be registered by following the International Caries Detection and Assessment System (ICDAS), which differentiates seven stages of caries development (from sound to early enamel lesion to extensive cavity) [27]. Gingival health will be evaluated using the Gingival Bleeding Index [28]. Participants will be examined in a supine position, with an illuminated mouth mirror and a CPI probe. The tooth status will be assessed by visual inspection, aided by tactile inspection if necessary. Duplicate examinations will be carried out on 10 % of the participants to assess intraexaminer reliability.

Before each oral examination, a questionnaire will be completed by the participants on a self-administered basis to gather information on their sociodemographic background and oral health knowledge, self-efficacy and behaviors. For oral health behaviors, participants will be asked how many times they snack and brush their teeth in a day. Their oral health self-efficacy will be measured by asking the participants whether they can “control snacks to once a day or less” and “brush teeth at least twice a day” under six scenarios, namely “when feeling down in the dumps,” “when busy and overloaded,” “when tired,” “when on a vacation,” “when it means stopping doing something that is enjoyable,” and “if there is a slip up, will the subject resume controlling” [29]. Participants are required to rate their control of oral health behaviors among “not at all,” “a little of the time,” “sometimes,” “most of the time” and “all the time.”

### Statistical analysis

Data will be analyzed using the computer software Statistical Package for Social Sciences (SPSS). Effectiveness of the intervention schemes over 6, 12 and 24 months will be evaluated on an intention-to-treat basis. All participants randomly assigned to each group will be included in the analysis, regardless of whether they receive the intervention or complete the study. If a participant is lost to follow-up, the last known outcome status will be carried forward.

Chi-square tests will be used for comparing distributions, whereas parametric or nonparametric methods, as appropriate, will be adopted for comparing means. Effect sizes (relative risk) and their 95 % confidence intervals (95 % CI) will be calculated and reported with *P* values. Multivariate analysis, namely ANCOVA (analysis of covariance) and multiple regressions, will be performed to evaluate the effects of various factors (intervention, school, counselor, participant’s sex, socioeconomic background and baseline caries experience) on the outcomes.

### Process evaluation and preliminary economic evaluation

A process evaluation will be included in this study for monitoring the recruitment, randomization, and implementation of the interventions. Using 15 % counseling sessions randomly selected, the MI fidelity will be measured by using the MITI scale. The interactions between the counselor and participants (in both modes of MI interventions) and among participants (in online group MI) will be analyzed. In addition, possible effect mediators such as “number of change talks,” “percentage of open questions” and “ratio of questions and reflections” will be recorded. In each intervention group, 20 participants will be interviewed to understand their acceptance and views toward the interventions.

The delivery of intervention mainly involves manpower cost. Hygienists’ time spent on each intervention will be recorded and a preliminary economic evaluation of the interventions will be conducted.

## Discussion

To the best of our knowledge, this study is the first attempt to introduce other mode of MI delivery, besides face-to-face individual counseling, into dental interventions. It taps into an unexplored area and is likely to generate useful evidence. Since online group MI is expected to be more convenient, accessible, and time efficient, it might address the practicality issues and pave the way for the application of MI in dental practice. The inclusion of online interventions into this study is also in line with the trend of Telehealth, in which information and communication technology is incorporated into health care delivery and is transforming the practice in many areas [30].

In this trial, randomization will be carried out at school level, instead of at individual level. Students in each participating school will receive the same intervention. This is to avoid intervention contamination, which is likely to happen if students in the same school are assigned to different groups. By randomly assigning the three schools from each district into three different groups and by monitoring the recruitment process, a balance will be reached in the characteristics of participants in the different groups with regard to their sociodemographic background and baseline behaviors. Since MI can rarely be completed in a single contact, follow-up phone calls will be made after the counseling session to assist the start of changes, explore solutions to the problems encountered, promote maintenance, avoid relapse and help re-establish change. Follow-up phone calls will not, however, be made in the HE group, so that the intervention reflects precisely the prevailing health education practice. The outcomes of the interventions will be evaluated by using psychological, behavioral and clinical measures at three points of time. This will allow us to interpret the results in a more complete manner.

Despite the benefits that online group MI may offer, it adds a layer of complexity to the process of counseling, as compared with the face-to-face individual sessions [14]. In a group setting, counselor may not be able to give individuals as much direct attention and floor time as in individual sessions [14]. Some group processes, such as spiral of negativity, if not guided properly, may inhibit a members’ motivation [14]. Face-to-face MI involves verbal exchange between the therapist and the participants, whereas online group MI in this study relies on text conversation. In addition, nonverbal cues are available in a face-to-face communication but are absent in online counseling. Although these factors may hamper to some extent the ability to form a therapeutical relationship in online counseling [31], because teens are already preferring to communicate via texting, online groups may be appealing in some other ways for them to interact with their peers comfortably [30]. Adequate training will be provided to counselors on the use of skills to facilitate group interaction and harness the power of group support, cohesion and momentum. The findings of this study will allow us to weigh the advantages and disadvantages of online group MI and shed some light on its potential uses in dental care.

MI has a strong empirical base in working with adolescents and young adults [18]. Since adolescents are developing an increased sense of independence and personal autonomy and are often resistant to direct advice, MI is considered as a useful method for working with this age group [18]. Findings of this study will provide much needed evidence for public health workers, dentists, and dental auxiliaries to choose effective and viable approaches in delivering behavioral interventions to adolescents. The findings will directly contribute to the prevention of common oral diseases (dental caries and periodontal diseases). Since unhealthy diet and poor personal hygiene are common risk factors accountable for many systemic diseases and conditions such as obesity, diabetes and infectious diseases, the intervention scheme identified in this study can also contribute to advancing adolescents’ general health.

## Trial status

Schools are being contacted. Participant recruitment will start soon.

## Abbreviations

MI: Motivational interviewing
HE: (prevailing) Health education
ICDAS: International Caries Detection and Assessment System
SPSS: Statistical Package for Social Sciences
